# Effect of Electrolyte Concentration and Pore Size on Ion Current Rectification Inversion

**DOI:** 10.1021/acsmeasuresciau.1c00062

**Published:** 2022-02-28

**Authors:** Dominik Duleba, Pallavi Dutta, Shekemi Denuga, Robert P. Johnson

**Affiliations:** School of Chemistry, University College Dublin, Belfield, Dublin 4 D04 V1W8, Ireland

**Keywords:** current rectification, nanopore, nanochannel, nanofluidic channel, ion transport, numerical simulation

## Abstract

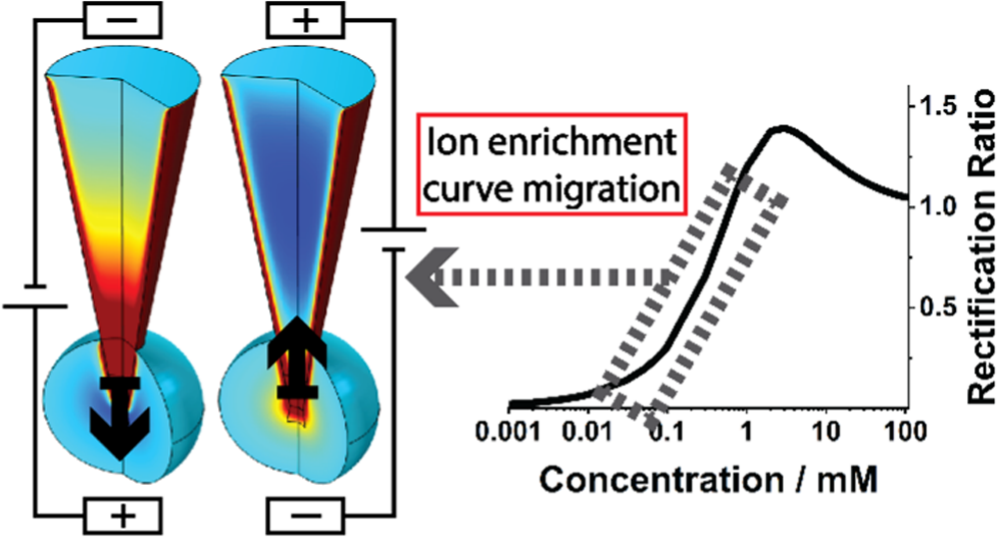

A thorough understanding of nanoscale transport properties is vital for the development and optimization of nanopore sensors. The thickness of the electrical double layers (EDLs) at the internal walls of a nanopore, as well as the dimensions of the nanopore itself, plays a crucial role in determining transport properties. Herein, we demonstrate the effect of the electrolyte concentration, which is inversely proportional to the EDL thickness, and the effect of pore size, which controls the extent of the electrical double layer overlap, on the ion current rectification phenomenon observed for conical nanopores. Experimental and numerical results showed that as the electrolyte concentration is decreased, the rectification ratio reaches a maximum, then decreases, and eventually inverts below unity. We also show that as the pore size is decreased, the rectification maximum and the inversion take place at higher electrolyte concentrations. Numerical investigations revealed that both phenomena occur due to the shifting of ion enrichment distributions within the nanopore as the electrolyte concentration or the pore size is varied.

## Introduction

The understanding of nanoscale transport properties is of significant interest in the effort to understand and optimize applications that utilize nanoscale spaces, such as nanopore sensors. The classical form of nanopore sensing is the resistive-pulse technique, which utilizes short-lived blockages of the nanopore as an analyte translocates, resulting in a detectable decrease in the steady-state current.^1^ This technique has found wide applications ranging from the detection of various analytes such as metal ions, molecules, nucleotides, and proteins, to DNA sequencing.^2−8^ Another more recent nanopore sensing platform is ionic current rectifying (ICR) nanopores.^9^ Nanopores with a charged surface and an asymmetry, which can be either an asymmetric geometry (such as a conical nanopore) or asymmetric surface charge distribution, have a non-Ohmic current–voltage curve, where the current measured at one potential is not the same as the current measured at the equal but opposite potential.^10−12^ Such a current–voltage curve is said to be rectified, and the rectification ratio (RR) is described as
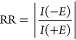
where *I*(−*E*) and *I*(+*E*) are the measured current values at a defined negative and positive potential, respectively.

**Figure 1:**
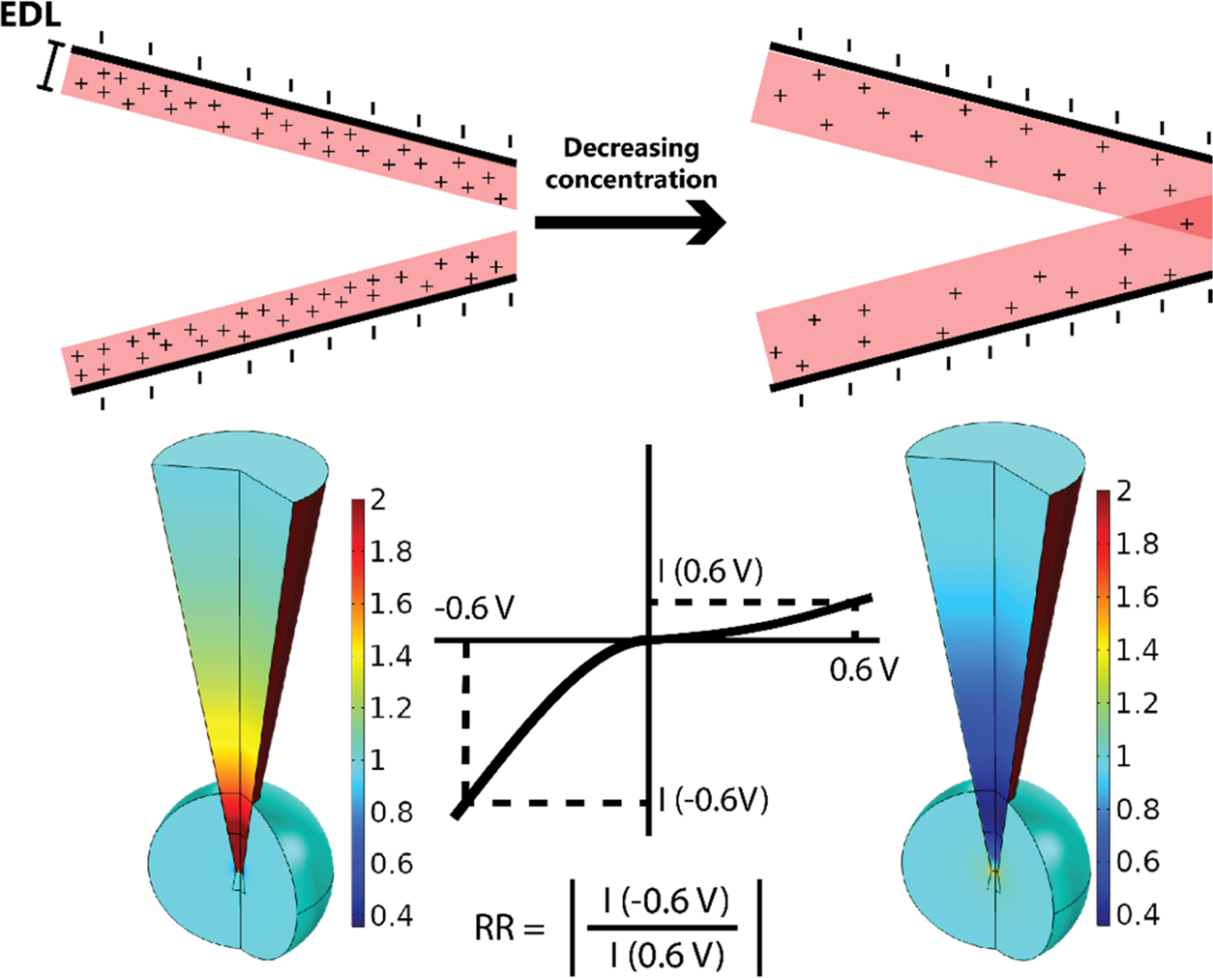
Thickening of the EDL as concentration is decreased and the rectified current–voltage curve, as well as the associated ion concentrations relative to the bulk concentration at the different potentials.

The rectification of ion transport in conical nanopores results primarily from the overlap of the electrical double layers (EDL) associated with the charged surface, with several potential models for phenomena described in the existing literature. For example, Woermann attributed the rectification to the changes of the transference numbers along the tip region (where the EDLs overlap) that, depending on the applied potential, cause a local ion enrichment leading to a high conductivity state or a local ion depletion leading to a low conductivity state.^13,14^ On the other hand, Siwy et al. described the electrostatic potential experienced by the cation along the length of the nanopore in terms of the overlapping EDLs, which, depending on the applied potential, can form an ion trap, leading to a low conductivity state (while the lack of an ion trap leads to a high conductivity state).^15,16^ Regardless of the model selected, it is evident that the EDL plays a critical role. The thickness of the electrical double layer, i.e., the extent to which it protrudes from nanopore walls, is characterized by the Debye length, which is inversely proportional to the concentration.^17^ Since the overlap of the EDL is critical for rectification, changes in the concentration can be expected to significantly affect the rectification of the nanopore (Figure 1).

It has been previously shown that after the initial increase of the rectification ratio with decreasing concentration (as would be expected from the subsequent increase in EDL thickness), the rectification ratio reaches a maximum, and then upon a further decrease in the concentration, the rectification ratio starts to decrease.^10,18−24^ The inversion of rectification below unity has also been numerically reported at very low electrolyte concentrations,^25−28^ but not previously shown experimentally.

However, rectification inversion below unity has been shown to take place with asymmetric electrolytes^21^ and with varying the scan rate,^26^ as well as pore angle and pore length.^29,30^ The postmaximum decrease of rectification has been associated with the emergence of significant concentration polarization at the tip leading to ion enrichment at the tip as well as an external ion depletion, which dominates the intrapore ion enrichment/depletion traditionally associated with ICR.^25,27−29^ Momotenko et al. showed that for ICR inversion below unity, the ion enrichment peaks at the pore mouth shifts inside or outside the pore depending on the applied polarity, changing the enrichment value at the narrowest part of the pore, controlling the extent of rectification.^28^ On the other hand, Yan et al. explained the decrease of rectification at low electrolyte concentrations and attributed it to external ion depletion and field focusing that become dominant once the pore selectivity is increased by decreasing the ion concentrations.^24^

Despite the theoretical predictions of an ICR inversion below unity through decreasing electrolyte concentration, the existence of such is yet to be confirmed experimentally, nor have the implications of the pore size on this inversion effect been explored in detail. Experimental studies of ICR inversion, as well as the implications of pore size on ICR inversion, are vital for progressing the design of nanopore sensors based on the ICR effect because these types of sensors rely on measuring changes to surface charge through host–guest binding events or similar interactions. To this end, we explore the inversion of rectification experimentally for the first time and, in addition, we demonstrate the effect of pore size on the position of the rectification maximum. Our work is supported by finite element simulations, which are used to confirm earlier theoretical studies, and then extended to rationalize the changing rectification maxima as the pore size and electrolyte concentration are varied.

## Experimental Methods

### Materials and Reagents

Potassium chloride (KCl) >99% was obtained from Fisher Scientific. Ag/AgCl electrodes were prepared in-house using silver wires obtained from Fisher Scientific. All solutions were prepared using Milli-Q-water from an Elga Purelab DV 35 water purification system. Glass capillaries with 1 mm outer diameter and 0.7 mm internal diameter (GQ100-70-7.5) were obtained from Sutter Instruments.

### Preparation of Nanopipettes and the Measurement of Rectification

Conical quartz nanopipettes were prepared using a P-2000 laser pipette puller from Sutter. Nanopipettes with 251 ± 100 nm (H575 F3 V60 D128 P100), 109 ± 20 nm (H580 F3 V55 D128 P110), 40 ± 4 nm (Line 1: H700 F4 V20 D170 P0, Line 2: H680 F4 V50 D170 P200), and 6 ± 1 nm (H750 F4 V40 D135 P180) pore radii were prepared using the programs outlined. The nanopipettes were backfilled with KCl solution using a microsyringe, and the current–voltage curves were measured using a two-electrode (Ag/AgCl and Ag/AgCl) setup as reported elsewhere,^31^ using a Biologic SP-200 potentiostat fitted with the ultra-low-current (ULC) option. Further technical details are provided in the Supporting Information. The sizes of the nanopipettes were determined based on their conductivity in 0.1 M electrolyte solution, as previously reported elsewhere.^31^

### Finite Element Simulations

Finite element analysis was carried out in the commercial software, COMSOL Multiphysics 6.0, where the Nernst–Plank equation (eq 1) is solved self-consistently with the Poisson equation (eq 2) and with the Navier–Stokes equation (eq 3) to obtain the concentration, potential, and the velocity/pressure distributions, respectively
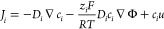
1where *J*_*i*_ denotes the ion flux, *D*_*i*_ the diffusion coefficient, and *z*_*i*_ the charge number of species *i*, *F* is the Faraday constant, *R* is the ideal gas constant, *T* is the temperature, Φ is the electric potential, and *u* is the flow velocity.
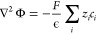
2where ϵ denotes the permittivity.
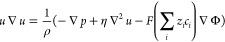
3where ρ is the fluid density and *p* is the pressure.

The Electrostatics (es), the Transport of Diluted Species (tds), and the Creeping Flow (spf) modules are used to incorporate the governing equations into the model. Incorporating the Navier–Stokes equations is important as it has a significant effect on the rectification ratios observed at electrolyte concentrations that correspond to the maximum rectification as shown in Figure S3 and as also reported by Ai et al.^25^ A two-dimensional (2D)-axisymmetric geometry with a 5 μm long pipette and a circular bulk solution that is 1 μm larger than the tip size is used. The tip of the nanopipette is described through a conical region with a half-cone angle of 10°, and a 5 nm tall cylindrical region, which is included to prevent sharp angles in the charged surface, which can cause numerical singularities. Due to its symmetry, this cylindrical region does not affect the observed rectification. The rectification ratios were calculated by applying +0.6/–0.6 V to the nanopore interior and extracting the currents passing through the pore. The normalized ion enrichment curves were obtained by extracting the average ion concentration results scaled by the bulk concentration along a 2D line on the central axisymmetric axis of the nanopipette. On this 2D line, the narrowest end of the conical nanopipette region is located at 0 nm, negative values denote the nanopipette interior, and positive values denote the nanopipette exterior. Further information regarding meshing and boundary conditions is available in the Supporting Information, alongisde the automatically generated COMSOL model report.

## Results and Discussion

**Figure 2:**
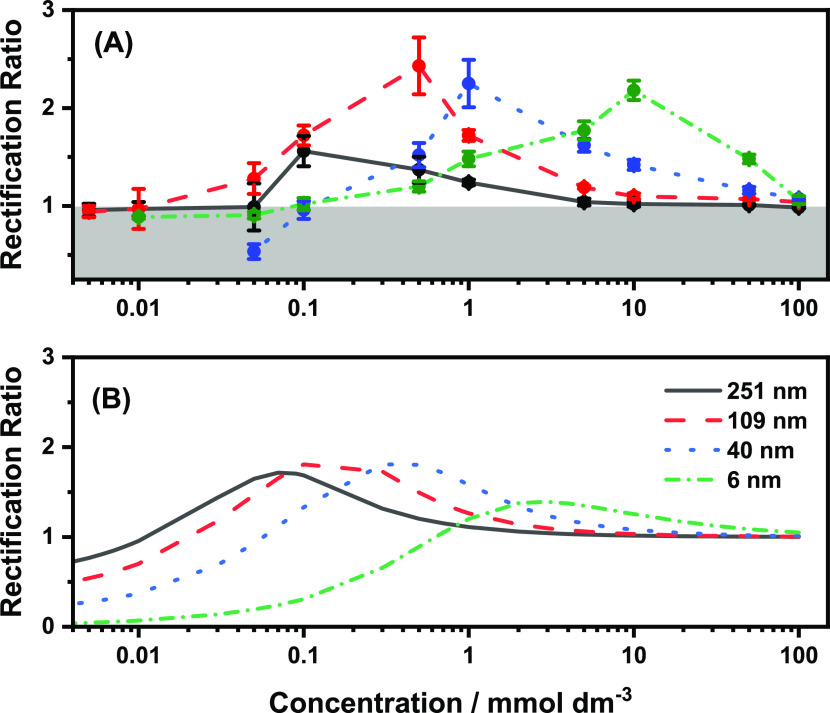
Rectification ratio is a function of both electrolyte concentration and pore size. The change in the rectification ratio as a function of electrolyte concentration for four different nanopore sizes obtained (A) experimentally and (B) numerically. Error bars are calculated as the standard error of measurements from a minimum of six nanopores. Representative current–voltage curves for each pore size and electrolyte concentration are shown in Figure S8.

Figure 2A shows the behavior of the rectification ratio (i.e., the extent to which the current deviates from ohmic behavior at a given applied potential) as a function of electrolyte concentration for four different nanopore sizes. At relatively high electrolyte concentrations, decreasing the electrolyte concentration leads to an increase in the rectification ratio as expected due to the thickening of the electrical double layer (EDL). However, a rectification ratio maximum is reached after which the rectification ratio decreases and eventually inverts below unity. As observable on both the experimental and simulated traces, the larger the radius of the nanopore, the lower the electrolyte concentration that is required to observe the rectification maxima. To reach the rectification maximum for a larger pore, an even smaller electrolyte concentration is needed, indicating that the phenomenon is a result of overlapping electrical double layers—which occurs at lower electrolyte concentrations for larger pores.

While an ICR maximum and the subsequent decrease of RR with decreasing electrolyte concentration has been predicted theoretically and shown experimentally,^10,18−24^ to the best of our knowledge, this is the first experimental report of ICR inversion below unity at very low electrolyte concentrations despite previous numerical predictions,^25−28^ and the first investigation of the effect of pore size on the position of the rectification maximum. We observed the greatest inversion below unity for the 40 nm pore (0.54 ± 0.08), with smaller inversions of 0.89 ± 0.02 and 0.94 ± 0.05 for the 6 and 109 nm pores, respectively. To confirm our experimental findings, finite element simulations were carried out (Figure 2B). Qualitative agreement between experiment and simulation with respect to the direction of change is present with the rectification maxima agreeing closer for the larger pores than for the smaller pores.

**Figure 3:**
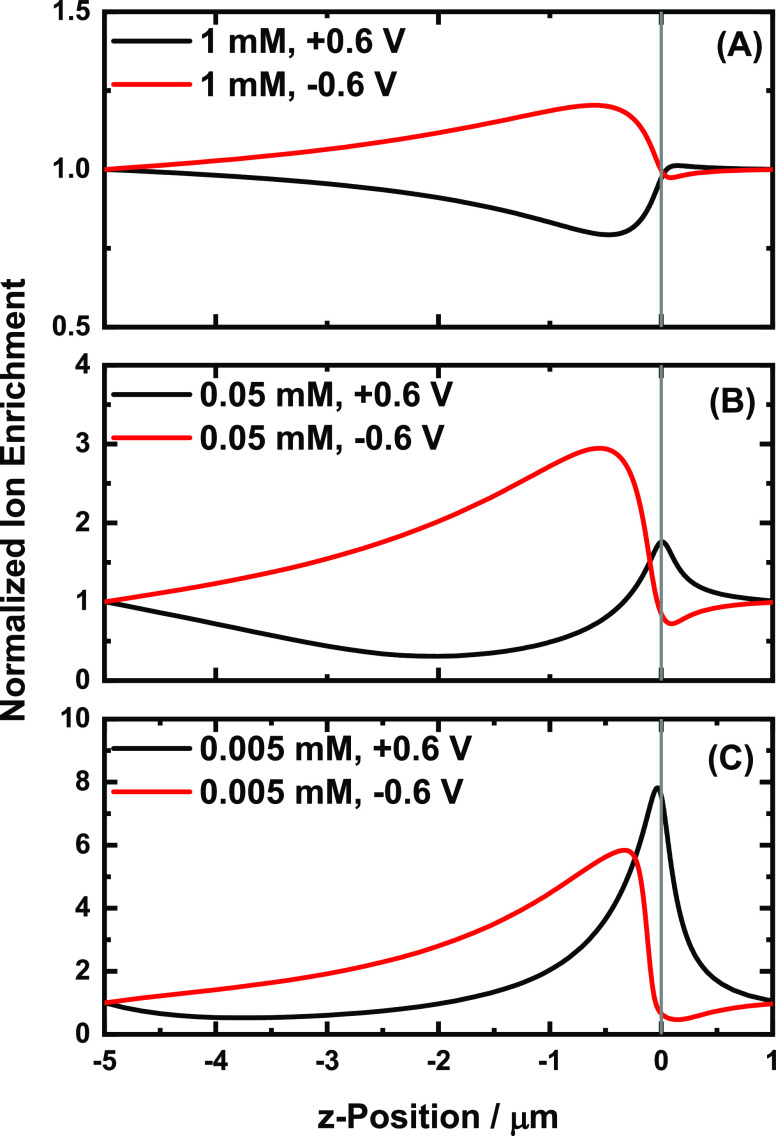
Normalized ion enrichment (*C*_av_/*C*_bulk_) values at (A) 1 mM, (B) 0.05 mM, and (C) 0.005 mM concentrations, corresponding to the prerectification maximum, postrectification maximum, and postrectification inversion regions, respectively. The normalized ion enrichment values were extracted from the central axisymmetric axis of the 109 nm pore. The gray reference line corresponds to the narrowest end of the conical region. The normalized cation and anion enrichment traces are shown in Figure S5.

In the classical rectifying region (i.e., at concentrations greater than that at which the rectification maximum is observed), previous reports^20,32^ show that ion depletion takes place within the pore when a positive potential is applied with respect to the pore exterior, and this results in a low conductivity state. Conversely, ion enrichment occurs when a negative potential is applied with respect to the pore exterior, and this results in a high conductivity state (Figure 3A). It is this difference in the conductivity deeper within the pore at the two equal but opposite potentials which results in rectification. In the immediate vicinity of the pore mouth, enrichment values are close to unity. On the other hand, in the postrectification maximum region (i.e., at low electrolyte concentrations) significant ion enrichment begins to arise at the pore mouth at both potentials (Figure 3B), and yet rectification (that is lower than that at the maximum) is still observed. Here, ion enrichment at the negative potential still takes place further inside the pore; however, enrichment contributions at the immediate vicinity of the pore mouth at the positive potential begin to contribute and decrease the rectification ratio. At very low electrolyte concentrations, where the rectification ratio inverts below unity, despite depletion occurring at the positive potential deeper inside the pore, the recorded currents will be larger at the positive potentials as contributions from the ion enrichments at the pore mouth become dominant (Figure 3C). These observations are in line with others reporting the emergence of concentration polarization at the pore mouth and its dominance over intrapore ion enrichment/depletion.^25,27−29^

In addition, as the electrolyte concentration is decreased, the pore selectivity for the cation increases in a sigmoidal fashion until saturation (transference number of 1) is reached in the inverse rectifying region (Figure S4). The selectivity for cations is greater at the positive potential than at the negative potential.^29^ This arises due to the influence of the applied potential on the ion concentrations and thus on the EDL thickness and pore selectivity.^29^ Ion enrichment at one potential will screen surface charges in the locality and hence decrease the EDL thickness and decrease the pore selectivity, while ion depletion at the other potential will locally increase the EDL thickness and increase the pore selectivity. At the same time, higher magnitude applied potentials increase the intensity of ion enrichment and ion depletion,^33^ enhancing the selectivity effect.

Interestingly, the pore mouth dominating conductivity was also reported in works examining the effect of the pore length on the rectification ratio.^29,30^ Zhang et al. showed that rectification can be reversed if the pore length is decreased and ion concentrations become more significant at the pore mouth.^29^ Ma et al. found that ultrashort nanopores with lower ion selectivity showed forward rectification, while ultrashort nanopores with high selectivity showed reverse rectification.^30^ The latter of which could be transformed into a forward rectifying pore as the pore length was increased. In the reverse rectifying pore, they also observed ion concentrations at the pore mouth dominating pore conductivity. These examples are highly relevant, as decreasing the nanopore length gives rise to a stronger electric field at the pore mouth that causes more significant ion enrichments, while in our case decreasing the electrolyte concentration increases the pore selectivity (by increasing the Debye length) and enhances concentration polarization at the pore mouth. As such decreasing, the pore length seems to influence transport properties similarly as decreasing the electrolyte concentration. Since the pore mouth dominates pore conductivity, we postulate that small shifts in the distributions of ion enrichments can have a significant effect on the observed rectification ratio.

**Figure 4:**
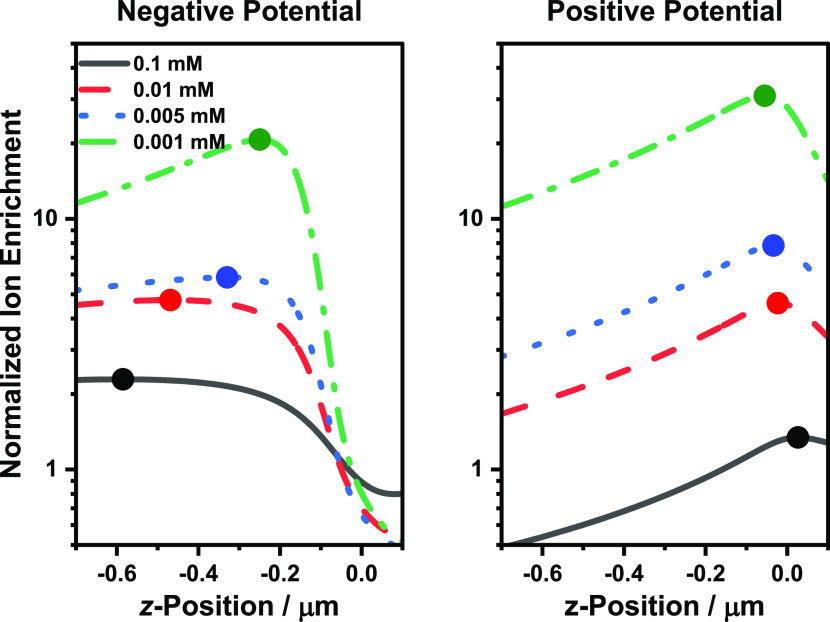
Normalized ion enrichment (*C*_av_/*C*_bulk_) peak shifts inside or outside the nanopore as a function of electrolyte concentration postrectification maximum. The normalized ion enrichment values were extracted from the central axisymmetric axis of the 109 nm nanopipette at different electrolyte concentrations. The point markers indicate the shifting location of the enrichment maximum. Note that a value <1 indicates depletion. The normalized cation and anion enrichment traces are shown in Figure S6.

The steady decrease in the rectification ratio as the concentration is decreased past the postrectification maxima can be explained by the shifting of the ion enrichment peaks as a function of concentration. We speculate that the shifting of the ion enrichment peak is related to the changing selectivity of the pore as the EDL increases with decreasing electrolyte concentrations. As shown in Figure 4, the enrichment peak at negative potentials moves outside the pore as the concentration is decreased, while the enrichment peak at the positive potential moves inside the pore as the concentration is decreased. The enrichment values at the pore mouth then change relative to each other such that the rectification ratio decreases. This shifting of the ion enrichment peaks, and the associated changes of the ion enrichment values at the pore mouth, also mean that at some concentration for a given pore size, the difference of the enrichment values at the pore mouth will reach a maximum. Thus, for each given pore size, there is a specific electrolyte concentration at which the relative highest and lowest conductivities are observed at the negative and positive potentials, respectively, resulting in the maximum rectification ratio.

**Figure 5:**
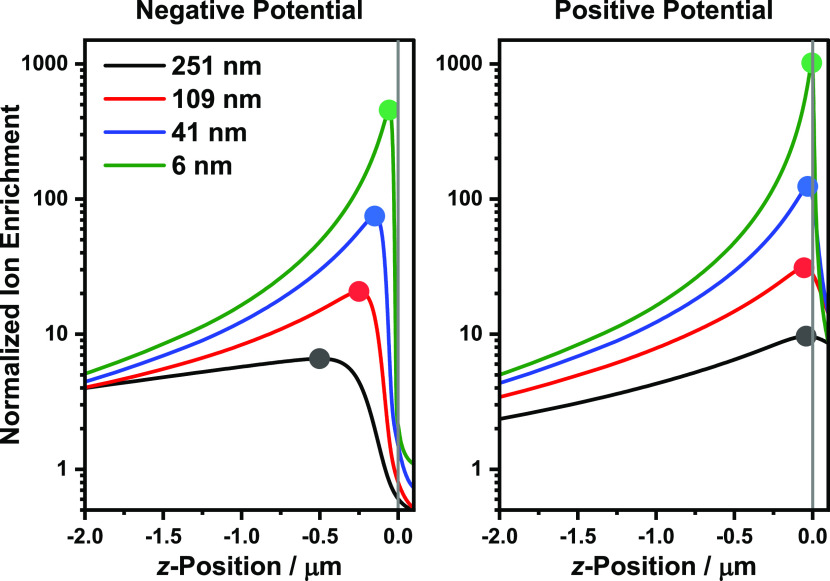
Normalized ion enrichment (*C*_av_/*C*_bulk_) peak shifts as the pore size is varied postrectification inversion (RR <1). The normalized ion enrichment values were extracted from the central axis symmetric axis at a 0.001 mM electrolyte concentration for the different pore sizes. The end of the conical nanopipette region is located at 0 nm. The point markers indicate the shifting location of the enrichment maximum. The normalized cation and anion enrichment traces are shown in Figure S7.

The position of the rectification maximum, for different sized pores, can be explained through the shifting of ion enrichment distributions as the pore size is varied. In this case, the enrichment maximum shifts outside the nanopipette as the pore size decreases at the negative potential, while the enrichment peak at the positive potential shifts only marginally (Figure 5). Since the enrichment peaks at the two potentials shift relative to each other, the enrichment values (and hence conductivities) in the pore mouth will also change in relation to each other at the two potentials, resulting in a different rectification.

At a specified electrolyte concentration, the enrichment peaks are positioned differently in pores of different sizes. It then follows that the extent to which ion enrichment with respect to the pore geometry needs to shift to exhibit maximum rectification is also dependent on the pore size. Since the position of the enrichment peak itself is also a function of concentration, each pore size will require a different electrolyte concentration to reach the maximum rectification, which accounts for the experimental and numerical observations.

## Conclusions

The inversion of rectification below unity, previously predicted numerically, is shown experimentally for the first time. Furthermore, it is demonstrated that as the pore size is increased, lower electrolyte concentrations are necessary to observe the rectification inversion. Finite element simulations are in qualitative agreement and reveal that the overall ion enrichment observable within the pore shifts as a function of both the concentration and pore size at negative potentials while shifting only for concentration at positive potentials. These mechanisms account for the behavior of the rectification inversion for both controllable parameters. Our results will assist in the understanding and development of ICR-based sensor technologies, which typically rely on nontrivial changes to surface charge upon the capture of a target analyte, such as proteins, on the internal walls of the nanopore.

## Supporting Information Available

Additional information on the finite element model’s meshing and boundary conditions, on the influence of electroosmotic flow, on the cation and anion normalized ion enrichment traces, on the second inversion of rectification observed at extremely low electrolyte concentrations, and on the experimental data collection. Sample current–voltage curves for each pore size and a COMSOL model report are also provided (PDF)
