# Supplementary material for: Effect of Electrolyte Concentration and Pore Size on Ion Current Rectification Inversion
Source: ACS Meas Sci Au. 2022 Feb 28;2(3):271–7. doi: 10.1021/acsmeasuresciau.1c00062 (PMC9204821; doi:10.1021/acsmeasuresciau.1c00062)
Supplement: Supplementary file 1 — tg1c00062_si_001.pdf [file tg1c00062_si_001.pdf]

# Supporting Information:

# The Effect of Electrolyte Concentration and Pore Size on Ion Current Rectification Inversion

Dominik Duleba[1†], Pallavi Dutta[1†], Shekemi Denuga[1], Robert P. Johnson[1] *

School of Chemistry, University College Dublin, Belfield, Dublin 4, Ireland

[†]These authors contributed equally.

* robert.johnson@ucd.ie

## 1. Meshing and Boundary Conditions

The meshing is constructed to be small near the tip opening and near the double layer (Figure S1b), while larger meshing is used in the bulk solution and in the further interior of the nanopipette (Figure S2a). The conical region, the double layers, and the space immediately outside the pore are further segregated into separate domains to allow better control of the mesh size. The mesh consists of triangular elements only. Since the tip size is changed between computations, it was important to adapt the mesh size at the tip to ensure that the meshing stays sufficiently fine without the computations becoming too costly. Mesh refinement studies were carried out and no change in rectification was observed upon further reduction of the mesh size, indicating that the mesh is sufficient to resolve the variations in the field variables solved for.

The bulk solution was constructed to be circular so that all bulk solution boundary elements are an equal distance from the tip mouth. Further increases in the length of the nanopipette and the radius of the bulk solutions were found to have no effect on the results of the model.

Boundary conditions were applied as shown in Figure S2. A constant concentration boundary was applied to the bulk solution boundaries and to the interior solution of the nanopipette, a potential boundary condition (+0.6 V or -0.6 V) was applied to the interior of the pipette and a ground boundary condition to the bulk solution. No slip conditions were applied to both the interior and exterior nanopipette walls, and no pressure gradient was applied between the interior and exterior solutions. Lastly, no flux and surface charge boundary conditions of $\sigma$ = 1 mC $m^{-2}$, were applied to both the inside and outside nanopipette walls.

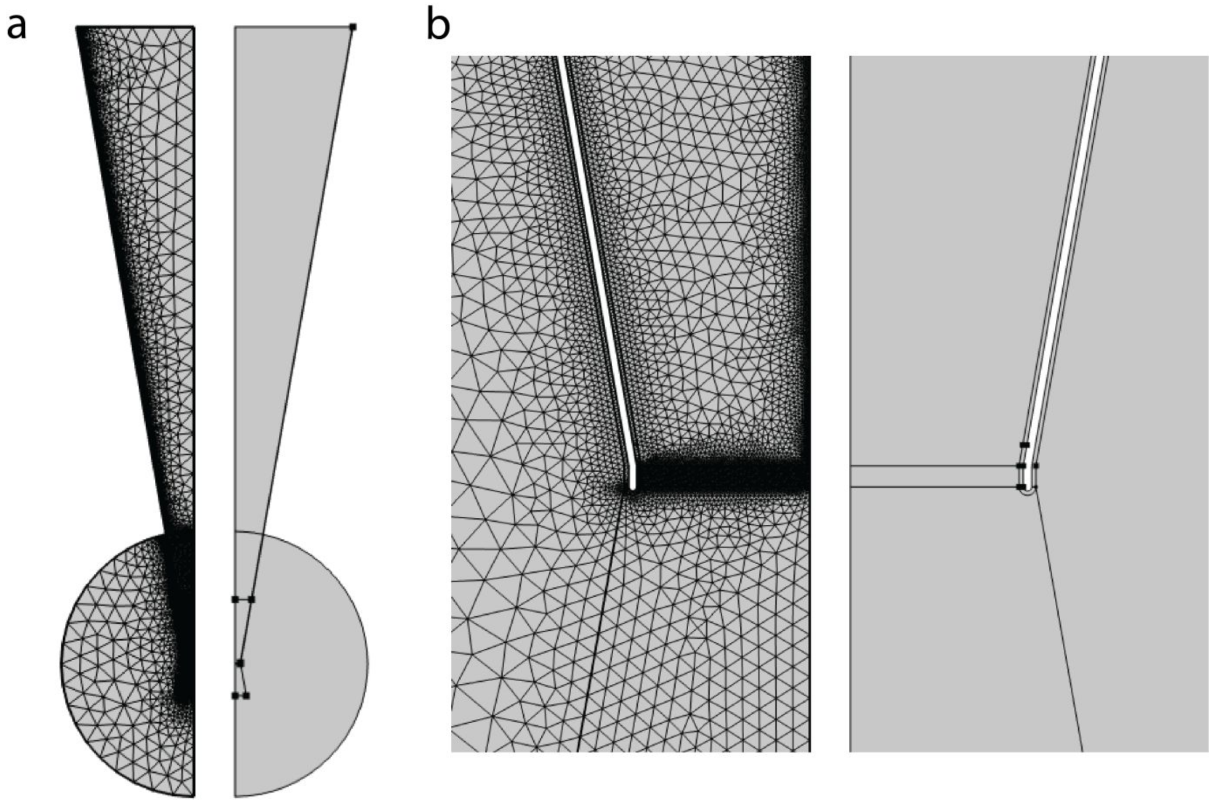


**Figure S1** The FEM geometry shown for the (a) whole model and (b) the tip region.

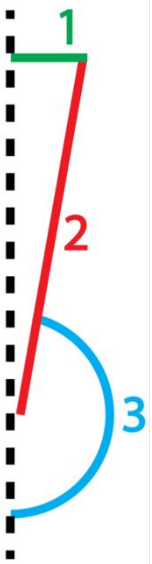


| Boundary no. | Boundary Description | Nernst-Planck eq. | Poisson eq. | Navier-Stokes eq. |
|---|---|---|---|---|
| 1 | Internal bulk solution | Constant Concentration $c = cbulk$ | Constant Potential (applied) $V = volt$ | Pressure = 0 |
| 2 | Pipette walls | No Flux $\boldsymbol{n} \cdot \nabla \boldsymbol{J}_i = 0$ | Surface Charge $\boldsymbol{n} \cdot \nabla\Phi = -\frac{\sigma}{e}$ | No Slip $\boldsymbol{u} = 0$ |
| 3 | External bulk solution | Constant Concentration $c = cbulk$ | Constant Potential (ground) $V = 0$ | Pressure = 0 |

**Figure S2** Boundary conditions applied in the FEM model.

## 2. The Effect of Electroosmotic Flow

Finite Element Analysis was carried out both with and without the Navier-Stokes equations which include the electroosmotic body force. As visible on Figure S3, the electroosmotic flow has a significant effect for the larger pores where the magnitude of the rectification ratio at the rectification maximum increases and where the peak maximum shifts to slightly lower concentrations. This effect is negligible for the smaller 6 nm pore, where the two curves are superimposed.

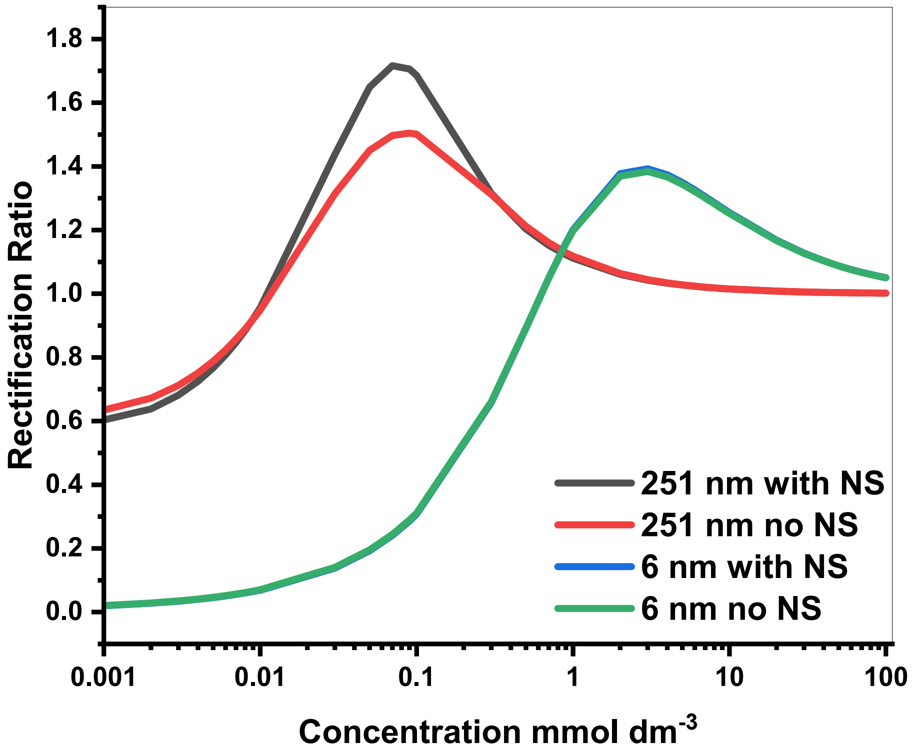


**Figure S3** The effect of including the Navier-Stokes equations for the largest and the smallest pores.

### 3. Transference numbers as a function of electrolyte concentration

Figure S4 shows the cation transference number of the pore as a function of electrolyte concentration.

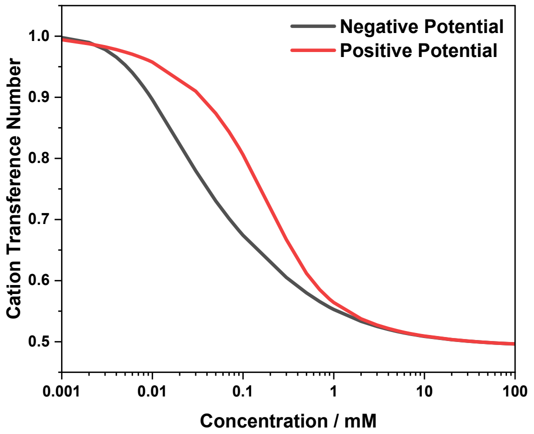


**Figure S4** The cation transference number as a function of concentration extracted from the results of the 109 nm pore.

## 4. Cation and anion traces

Figure S5 shows the cation and anion traces associated with Figure 3 in the main paper. It shows how the cation and anion traces become significantly different as the EDL length increases.

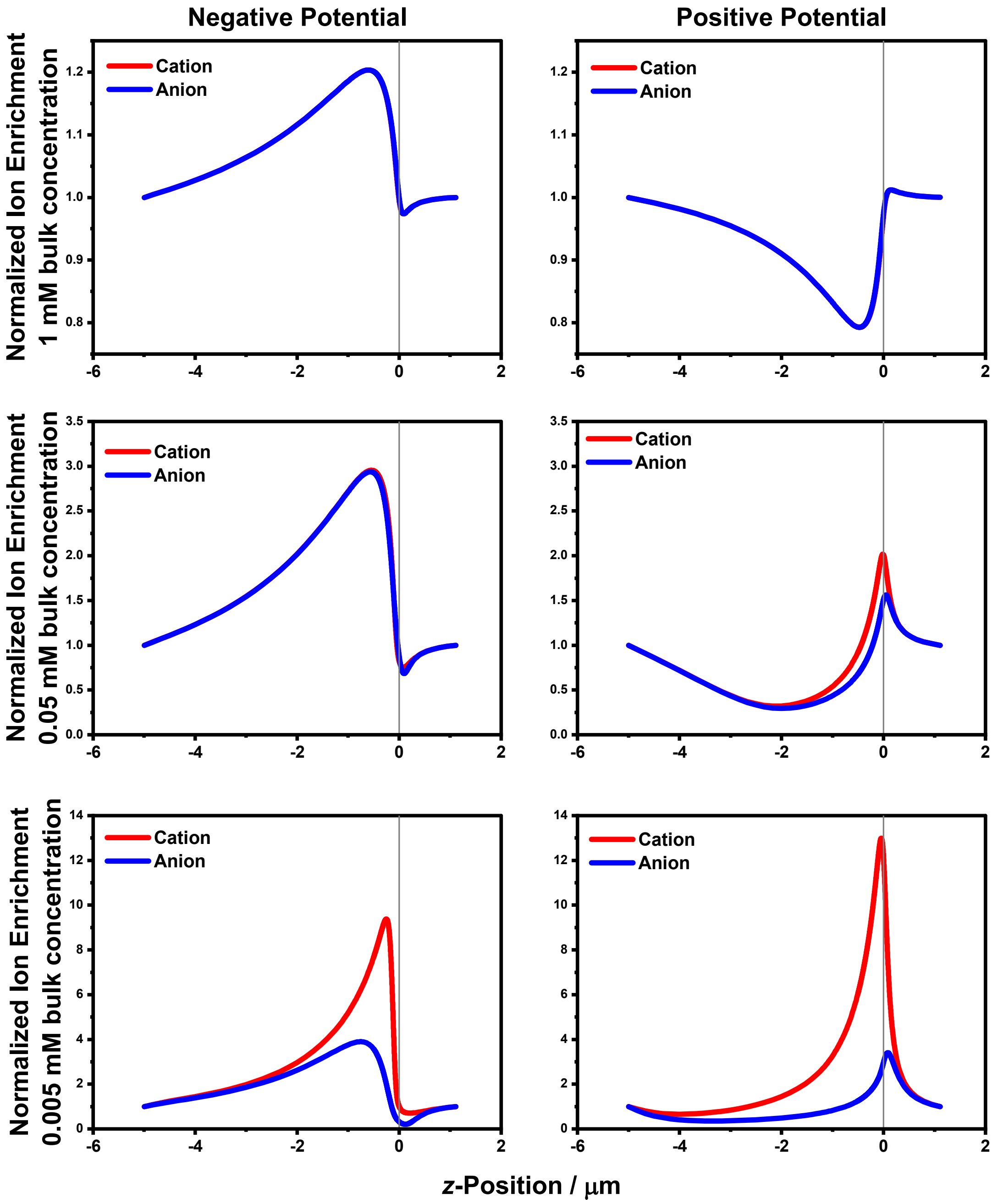

**Figure S5** Normalized ion enrichment curves for the cations and anions corresponding the curves shown in Figure 3. The normalized ion enrichment values were extracted from the central axissymmetric axis of the 109 nm pore.

Figure S6 shows that the individual cation and anion enrichment also shifts as a function of electrolyte concentration. At the negative potential, both the cation and anion enrichment peaks shift further outside the pore as the electrolyte concentration is lowered. On the other hand, at the positive potential, the cation enrichment peak shifts inside the pore, while the anion enrichment peak shifts outside as the electrolyte concentration is decreased.

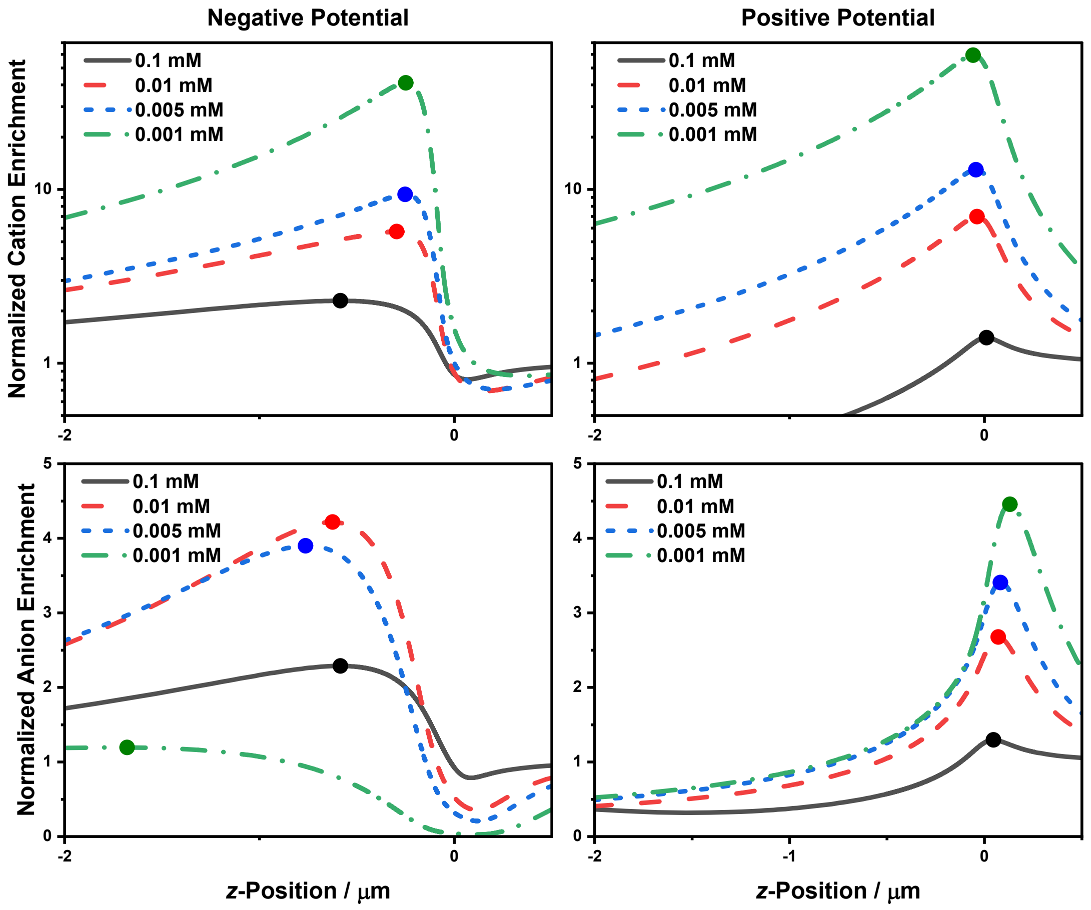


**Figure S6** Normalized ion enrichment curves for the cations and anions corresponding the curves shown in Figure 4. The normalized ion enrichment values were extracted from the central axissymmetric axis of the 109 nm pore.

Figure S7 shows that the cation and anion ion enrichment traces shift with the pore size. The cation enrichment peaks shift outside the pipette as the pore size is decreased at both the positive and negative potentials. On the other hand, the anion enrichment peak shifts further inside the pipette as the pore size is decreased at both the positive and negative potentials.

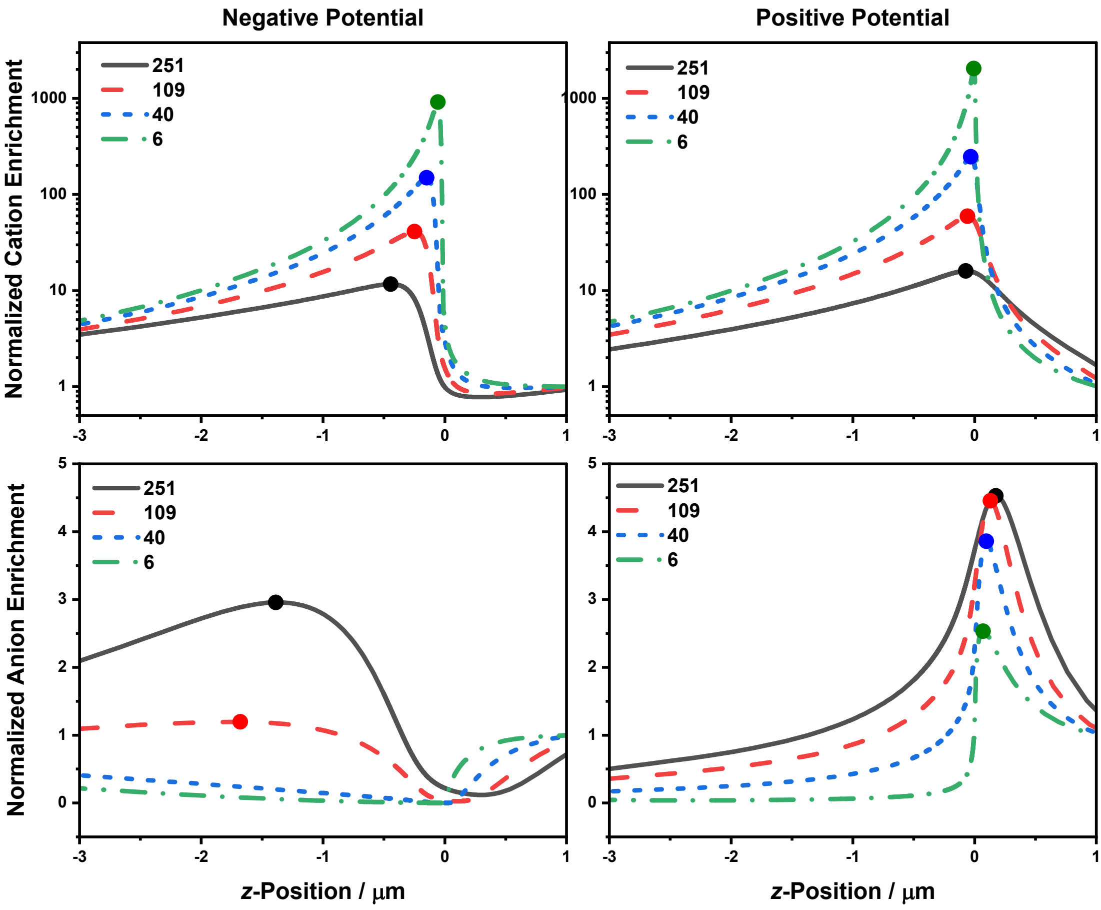


**Figure S7** Normalized ion enrichment curves for the cations and anions corresponding the curves shown in Figure 5. The normalized ion enrichment values were extracted from the central axissymmetric axis of nanopipette at a 0.001 mM electrolyte concentration for the different pore sizes.

## 5. Second inversion of rectification

A second inversion of rectification, similar to that reported by Momotenko et al., was also observed by us numerically, however, the experimental observation of this second rectification was beyond our measurement capabilities. The second inversion was predicted to occur at an electrolyte concentration of around 0.0001 mM for the 6 nm pores, however, the noise associated with the measurement of the current-voltage curves at these concentrations would lead to a large uncertainty in the extracted RR which would not allow the reliable experimental observation of the second rectification. Furthermore, at such low concentrations, the uncertainty in the concentration of the prepared solutions also becomes significant.

## 6. Sample Current-Voltage Curves

A Biologic SP-200 potentiostat was used for collecting the current-voltage curves. Ultra-low current option at a high-speed scan is employed; a filter bandwidth of 50 kHz is used during data collection. Noise is further reduced by numerically filtering the data after their acquisition using a moving average filter with a windows size of 11 points in EC-lab V11.34. The parameter settings for the recorded current-voltage curves includes 3 scans taken at a scan rate of 0.1 V/s within a potential window of -0.6 to +0.6 V.

Figure S8 shows samples of the current-voltage curves for each pore size at the electrolyte concentration where maximum rectification was observed. It is important to point out that the noise is the smallest for the 40 nm pore at maximum rectification. This arises since the noise is proportional to the pore size (larger pores carry larger currents) but inversely proportional to the electrolyte concentration (smaller electrolyte concentrations lead to smaller current magnitudes). Since larger pores have their rectification maximum at lower electrolyte concentrations, the noise at the rectification maximum is minimized for the 40 nm pore.

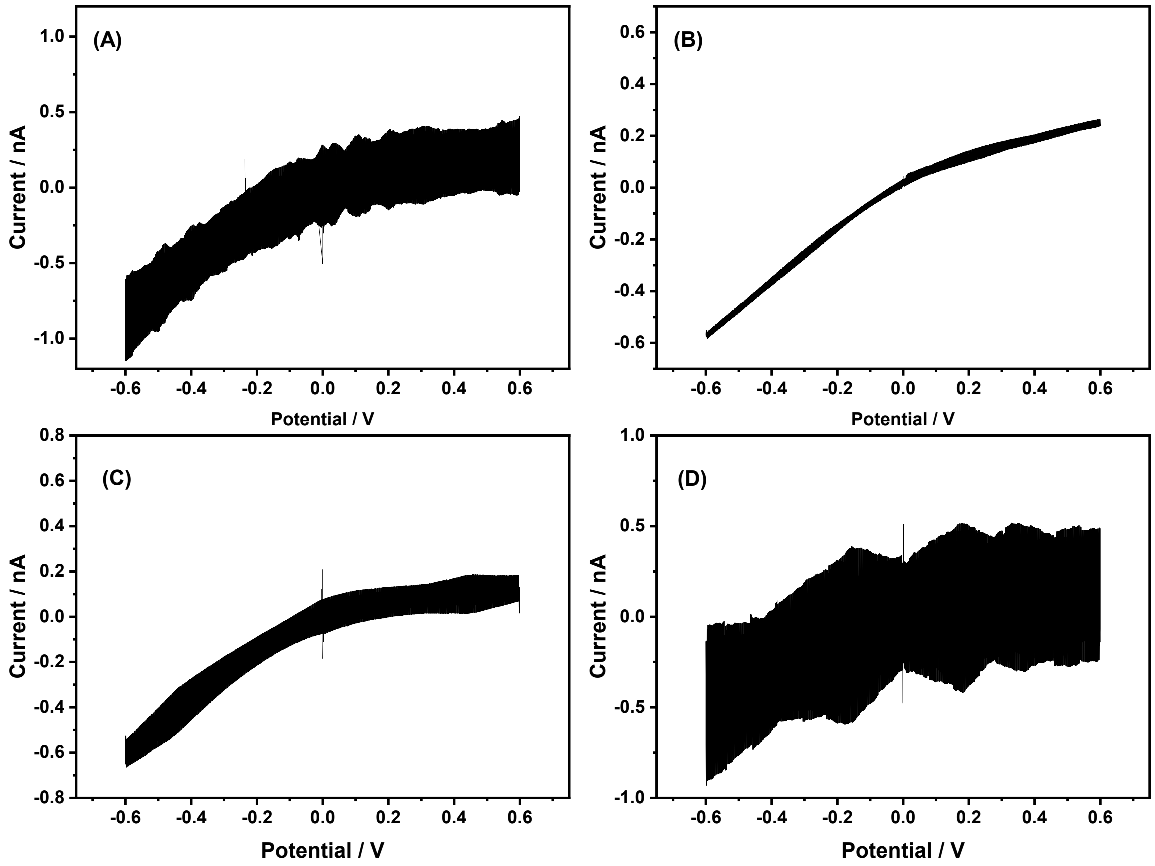


**Figure S8** Sample Current-Voltage curves showing (A) a 6 nm pore at 10 mM concentration, (B) a 40 nm pore at 1 mM concentration, (C) a 251 nm pore at 0.1 mM concentration, and (D) a 109 nm pore at 0.5 mM concentration.

## 7. Comsol Model Report

# Model: The Effect of Electrolyte Concentration and Pore Size on Ion Current Rectification Inversion

# Contents


**1 Global Definitions** .......... **3**

1.1 Parameters .......... 3

1.2 Shared Properties .......... 3

**2 Component 1** .......... **4**

2.1 Definitions .......... 4

2.2 Geometry 1 .......... 4

2.3 Materials .......... 10

2.4 Electrostatics .......... 11

2.5 Transport of Diluted Species .......... 18

2.6 Creeping Flow 2 .......... 25

2.7 Multiphysics .......... 33

2.8 Mesh 1 .......... 37

# Global Definitions

### GLOBAL SETTINGS

| | |
|---|---|
| Version | COMSOL Multiphysics 6.0 (Build: 312) |

### USED PRODUCTS

| |
|---|
| Electrochemistry Module |
| COMSOL Multiphysics |

### COMPUTER INFORMATION

| | |
|---|---|
| CPU | Intel64 Family 6 Model 165 Stepping 5, 8 cores |
| Operating system | Windows 10 |

## PARAMETERS

### PARAMETERS 1

| Name | Expression | Value | Description |
|---|---|---|---|
| surfCharge | -0.001[C/(m*m)] | $-0.001\ C/m^2$ | Glass Wall Surface Charge |
| volt | 1 [V] | 1 V | Applied Potential |
| zK | 1 | 1 | Charge Number of Cation |
| zCl | -1 | −1 | Charge Number of Anion |
| diffCl | 2.032e-5[cm^2/s] | $2.032E-9\ m^2/s$ | chloride diffusion coefficient |
| diffK | 1.957e-5[cm^2/s] | $1.957E-9\ m^2/s$ | potassium diffusion coefficient |
| cbulk | 0.01[M] | $10\ mol/m^3$ | Bulk Concentration |
| pipH | 5[um] | 5E−6 m | pipette height |
| theta | 10[deg] | 0.17453 rad | pipette cone angle |
| poreR | 109[nm] | 1.09E−7 m | pipette radius |
| cylH | 5[nm] | 5E−9 m | tip cylinder height |
| bathR | poreR +1[um] | 1.109E−6 m | external bath radius |
| wall | 2[nm] | 2E−9 m | pipette glass wall thickness |
| epsilon | 78.5 | 78.5 | electrolyte permittivity |
| T | 298 | 298 | Temperature |
| DLmesh | 1[nm] | 1E−9 m | double layer mesh distance |
| pipR | (pipH - cylH)*tan(theta) + poreR | 9.8975E−7 m | top pipette width |

## SHARED PROPERTIES

## Default Model Inputs

| | |
|---|---|
| Tag | cminpt |

# Component 1

SETTINGS

| Description | Value |
| --- | --- |
| Unit system | Same as global system (SI) |

## DEFINITIONS

## Coordinate Systems

### Boundary System 1

| | |
| --- | --- |
| Coordinate system type | Boundary system |
| Tag | sys1 |

COORDINATE NAMES

| First | Second | Third |
| --- | --- | --- |
| t1 | to | n |

## GEOMETRY 1

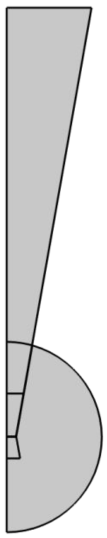

*Geometry 1*

UNITS

| | |
| --- | --- |
| Length unit | m |
| Angular unit | deg |

GEOMETRY STATISTICS

| Description | Value |
| --- | --- |
| Space dimension | 2 |
| Number of domains | 13 |
| Number of boundaries | 42 |
| Number of vertices | 30 |

## Conical Pipette Region (pol1)

### OBJECT TYPE

| Description | Value |
|---|---|
| Type | Solid |

### COORDINATES

| Description | Value |
|---|---|
| Data source | Table |

### COORDINATES

| r (m) | z (m) |
|---|---|
| poreR | cylH |
| (pipH-cylH)*tan(theta)+poreR | pipH |
| 0 | pipH |
| 0 | cylH |

## Cylinder (r1)

### POSITION

| Description | Value |
|---|---|
| Position | {0, 0} |

### SIZE

| Description | Value |
|---|---|
| Width | poreR |
| Height | cylH |

## Bulk Solution (c1)

### POSITION

| Description | Value |
|---|---|
| Position | {0, -2E-9} |

### ROTATION ANGLE

| Description | Value |
|---|---|
| Rotation | 270 |

### SIZE AND SHAPE

| Description | Value |
|---|---|
| Radius | bathR |
| Sector angle | 180 |

## Glass Wall (pol2)

### OBJECT TYPE

| Description | Value |
|---|---|
| Type | Solid |

COORDINATES

| Description | Value |
|---|---|
| Data source | Table |

COORDINATES

| r (m) | z (m) |
|---|---|
| poreR | 0 |
| poreR+wall | 0 |
| poreR+wall | cylH |
| poreR+DLmesh+(pipH-cylH)*tan(theta) | pipH |
| poreR+(pipH-cylH)*tan(theta) | pipH |
| poreR | cylH |

### Circular Glass Wall (c2)

POSITION

| Description | Value |
|---|---|
| Position | {1E-9 + poreR, 0} |

ROTATION ANGLE

| Description | Value |
|---|---|
| Rotation | 180 |

SIZE AND SHAPE

| Description | Value |
|---|---|
| Radius | 1E-9 |
| Sector angle | 180 |

### Tip DL for meshing (pt1)

POINT

| Description | Value |
|---|---|
| Point coordinate | {1.0800000000000001E-7, 0} |

### Inner Cylinder DL for meshing (pt2)

POINT

| Description | Value |
|---|---|
| Point coordinate | {1.0800000000000001E-7, 5.0E-9} |

### Pipette top DL for meshing (pt3)

POINT

| Description | Value |
|---|---|
| Point coordinate | {9.887532686387823E-7, 4.9999999999999996E-6} |

### Curved tip DL for meshing (c3)

#### POSITION

| Description | Value |
|---|---|
| Position | {(wall/2) + poreR, 0} |

#### ROTATION ANGLE

| Description | Value |
|---|---|
| Rotation | 180 |

#### SIZE AND SHAPE

| Description | Value |
|---|---|
| Radius | (wall/2) + DLmesh |
| Sector angle | 180 |

### Outer cylinder DL for meshing (pt4)

#### POINT

| Description | Value |
|---|---|
| Point coordinate | {1.1200000000000001E-7, 5.0E-9} |

### Outer Pipette top DL (pt5)

#### POINT

| Description | Value |
|---|---|
| Point coordinate | {9.927532686387824E-7, 4.9999999999999996E-6} |

### Cone bottom, wall, fix low element quality (pt6)

#### POINT

| Description | Value |
|---|---|
| Point coordinate | {1.0988163490354234E-7, 1.0E-8} |

### Cone bottom, DL, fix low element quality (pt7)

#### POINT

| Description | Value |
|---|---|
| Point coordinate | {1.0888163490354234E-7, 1.0E-8} |

### Lower cone region for finer meshing r=0 (pt12)

#### POINT

| Description | Value |
|---|---|
| Point coordinate | {0, 5.05E-7} |

### Lower cone region for finer meshing DL (pt13)

POINT

| Description | Value |
|---|---|
| Point coordinate | {1.9616349035423253E-7, 5.05E-7} |

### Lower cone region for finer meshing, wall (pt16)

POINT

| Description | Value |
|---|---|
| Point coordinate | {1.9716349035423252E-7, 5.05E-7} |

### Barrel top, wall, fix low element quality (pt8)

POINT

| Description | Value |
|---|---|
| Point coordinate | {1.0900000000000001E-7, 0} |

### Barrel top, DL, fix low element quality (pt9)

POINT

| Description | Value |
|---|---|
| Point coordinate | {1.0800000000000001E-7, 0} |

### Barrel bottom, wall, fix low element quality (pt10)

POINT

| Description | Value |
|---|---|
| Point coordinate | {1.0900000000000001E-7, 5.0E-9} |

### Barrel bottom, DL, fix low element quality (pt11)

POINT

| Description | Value |
|---|---|
| Point coordinate | {1.0800000000000001E-7, 5.0E-9} |

### Bulk below pipette (meshing) r=0 (pt14)

POINT

| Description | Value |
|---|---|
| Point coordinate | {0, -2.5000000000000004E-7} |

### Bulk below pipette (meshing) (pt15)

POINT

| Description | Value |
|---|---|
| Point coordinate | {1.5608174517711627E-7, -2.5000000000000004E-7} |

## MATERIALS

### Water, liquid

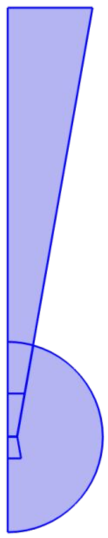

*Water, liquid*

#### SELECTION

| | |
|---|---|
| Geometric entity level | Domain |
| Selection | Geometry geom1: Dimension 2: All domains |

#### MATERIAL PARAMETERS

| **Name** | **Value** | **Unit** |
|---|---|---|
| Dynamic viscosity | eta(T) | Pa·s |
| Density | rho(T) | kg/m$^3$ |
| Relative permittivity | 80 | 1 |

#### BASIC

| **Description** | **Value** |
|---|---|
| Coefficient of thermal expansion | {{alpha_p(T), 0, 0}, {0, alpha_p(T), 0}, {0, 0, alpha_p(T)}} |
| Bulk viscosity | muB(T) |
| Dynamic viscosity | eta(T) |
| Ratio of specific heats | gamma_w(T) |
| Electrical conductivity | {{5.5e-6[S/m], 0, 0}, {0, 5.5e-6[S/m], 0}, {0, 0, 5.5e-6[S/m]}} |
| Heat capacity at constant pressure | Cp(T) |
| Density | rho(T) |
| Thermal conductivity | {{k(T), 0, 0}, {0, k(T), 0}, {0, 0, k(T)}} |
| Speed of sound | cs(T) |
| Relative permittivity | {{80, 0, 0}, {0, 80, 0}, {0, 0, 80}} |

## ELECTROSTATICS

#### USED PRODUCTS

| COMSOL Multiphysics |
|---|

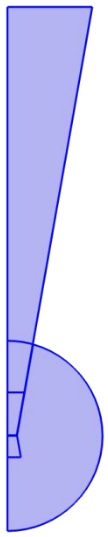

*Electrostatics*

SELECTION

| | |
|---|---|
| Geometric entity level | Domain |
| Selection | Geometry geom1: Dimension 2: All domains |

EQUATIONS

$$\nabla \cdot \mathbf{D} = \rho_{\mathrm{v}}$$

$$\mathbf{E} = -\nabla V$$

## Interface Settings

### Discretization

SETTINGS

| Description | Value |
|---|---|
| Electric potential | Quadratic |

### Manual Terminal Sweep Settings

SETTINGS

| Description | Value |
|---|---|
| Use manual terminal sweep | Off |
| Reference impedance | 50[ohm] |

### Charge Conservation 1

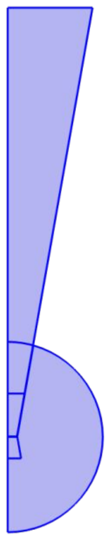

*Charge Conservation 1*

SELECTION

| | |
|---|---|
| Geometric entity level | Domain |
| Selection | Geometry geom1: Dimension 2: All domains |

EQUATIONS

$$\mathbf{E} = -\nabla V$$
$$\nabla \cdot (\epsilon_0 \epsilon_r \mathbf{E}) = \rho_v$$

### Constitutive Relation D-E

SETTINGS

| Description | Value |
|---|---|
| Dielectric model | Relative permittivity |
| Relative permittivity | From material |

### Coordinate System Selection

SETTINGS

| Description | Value |
|---|---|
| Coordinate system | Global coordinate system |

PROPERTIES FROM MATERIAL

| Property | Material | Property group |
|---|---|---|
| Relative permittivity | Water, liquid | Basic |

## Axial Symmetry 1

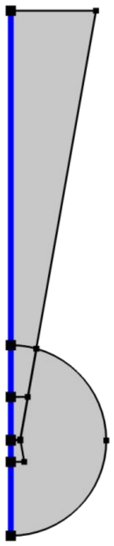

*Axial Symmetry 1*

SELECTION

| | |
|---|---|
| Geometric entity level | Boundary |
| Selection | Geometry geom1: Dimension 1: All boundaries |

## Zero Charge 1

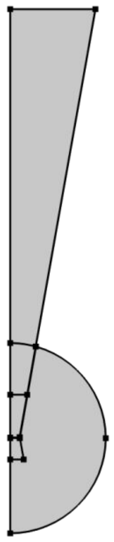

*Zero Charge 1*

SELECTION

| | |
|---|---|
| Geometric entity level | Boundary |
| Selection | Geometry geom1: Dimension 1: All boundaries |

EQUATIONS

$$\mathbf{n} \cdot \mathbf{D} = 0$$

## Initial Values 1

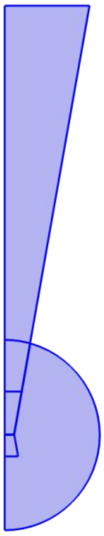

*Initial Values 1*

SELECTION

| | |
|---|---|
| Geometric entity level | Domain |
| Selection | Geometry geom1: Dimension 2: All domains |

SETTINGS

| Description | Value |
|---|---|
| Electric potential | 0 |

## Space Charge Density 1

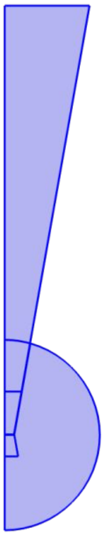

*Space Charge Density 1*

SELECTION

| | |
|---|---|
| Geometric entity level | Domain |
| Selection | Geometry geom1: Dimension 2: All domains |

EQUATIONS

$$\nabla \cdot \mathbf{D} = \rho_{\mathrm{v}}$$

### Coordinate System Selection

SETTINGS

| Description | Value |
|---|---|
| Coordinate system | Global coordinate system |

## Ground 1

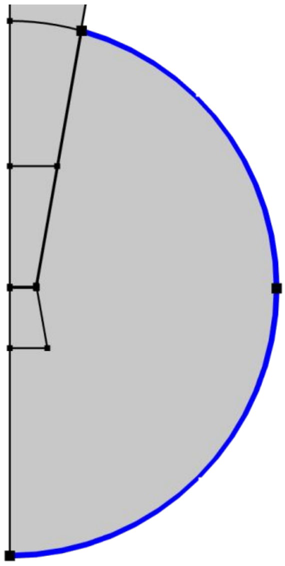

*Ground 1*

SELECTION

| | |
|---|---|
| Geometric entity level | Boundary |
| Selection | Geometry geom1: Dimension 1: Boundaries 34, 41–42 |

EQUATIONS

$$V = 0$$

### Constraint Settings

SETTINGS

| Description | Value |
|---|---|
| Apply reaction terms on | All physics (symmetric) |
| Use weak constraints | On |

## Applied Potential

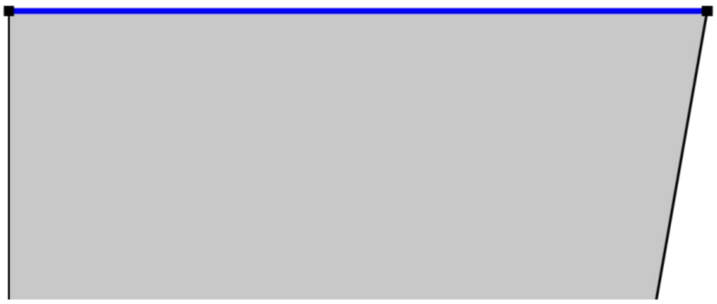

*Applied Potential*

SELECTION

| | |
|---|---|
| Geometric entity level | Boundary |
| Selection | Geometry geom1: Dimension 1: Boundaries 12, 33 |

EQUATIONS

$$V = V_0$$

### Electric Potential

SETTINGS

| Description | Value |
|---|---|
| Electric potential | volt |

### Constraint Settings

SETTINGS

| Description | Value |
|---|---|
| Apply reaction terms on | All physics (symmetric) |
| Use weak constraints | On |

## Surface Charge Density

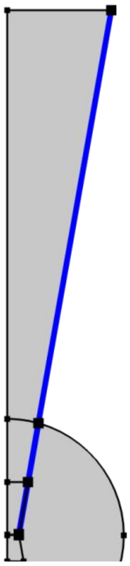

*Surface Charge Density*

SELECTION

| | |
|---|---|
| Geometric entity level | Boundary |
| Selection | Geometry geom1: Dimension 1: Boundaries 19–22, 24, 30, 32, 37, 39 |

EQUATIONS

$$\mathbf{n} \cdot (\mathbf{D}_1 - \mathbf{D}_2) = \rho_s$$

### Surface Charge Density

SETTINGS

| Description | Value |
|---|---|
| Surface charge density | surfCharge |

### Coordinate System Selection

SETTINGS

| Description | Value |
|---|---|
| Coordinate system | Global coordinate system |

## TRANSPORT OF DILUTED SPECIES

USED PRODUCTS

| |
|---|
| Electrochemistry Module |
| COMSOL Multiphysics |

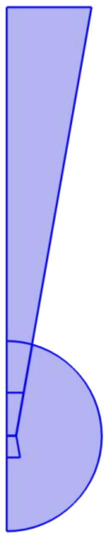

*Transport of Diluted Species*

SELECTION

| | |
|---|---|
| Geometric entity level | Domain |
| Selection | Geometry geom1: Dimension 2: All domains |

EQUATIONS

$$\nabla\cdot\mathbf{J}_i+\mathbf{u}\cdot\nabla c_i=R_i$$
$$\mathbf{J}_i=-D_i\nabla c_i-z_iu_{m,i}Fc_i\nabla V$$

## Interface Settings

### Discretization

SETTINGS

| Description | Value |
|---|---|
| Concentration | Quadratic |

SETTINGS

| Description | Value |
|---|---|
| Equation form | Study controlled |

### Transport Mechanisms

SETTINGS

| Description | Value |
|---|---|
| Convection | On |
| Migration in electric field | On |
| Mass transfer in porous media | Off |

## Transport Properties 1

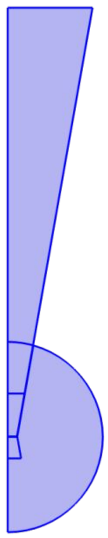

*Transport Properties 1*

### SELECTION

| | |
|---|---|
| Geometric entity level | Domain |
| Selection | Geometry geom1: Dimension 2: All domains |

### EQUATIONS

$$\nabla \cdot \mathbf{J}_i + \mathbf{u} \cdot \nabla c_i = R_i$$

$$\mathbf{J}_i = -D_i \nabla c_i - z_i u_{m,i} F c_i \nabla V$$

### SETTINGS

| Description | Value |
|---|---|
| Velocity field | Velocity field (rfd1) |

## Diffusion

### SETTINGS

| Description | Value |
|---|---|
| Source | Material |
| Material | Water, liquid (mat1) |
| Diffusion coefficient | User defined |
| Diffusion coefficient | {{diffK, 0, 0}, {0, diffK, 0}, {0, 0, diffK}} |
| Diffusion coefficient | User defined |
| Diffusion coefficient | {{diffCl, 0, 0}, {0, diffCl, 0}, {0, 0, diffCl}} |

## Migration in Electric Field

### SETTINGS

| Description | Value |
|---|---|
| Mobility | Nernst - Einstein relation |
| Charge number | {1, -1} |

### Coordinate System Selection

SETTINGS

| Description | Value |
|---|---|
| Coordinate system | Global coordinate system |

### Model Input

SETTINGS

| Description | Value |
|---|---|
| Temperature | User defined |
| Temperature | T |

## Axial Symmetry 1

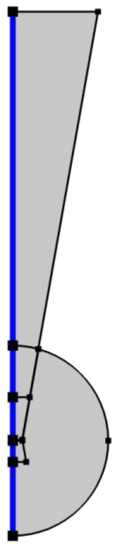

*Axial Symmetry 1*

SELECTION

| | |
|---|---|
| Geometric entity level | Boundary |
| Selection | Geometry geom1: Dimension 1: All boundaries |

### No Flux 1

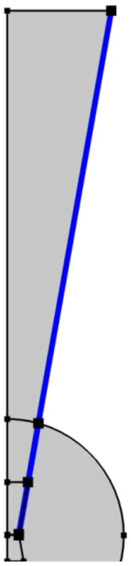

*No Flux 1*

SELECTION

| | |
|---|---|
| Geometric entity level | Boundary |
| Selection | Geometry geom1: Dimension 1: All boundaries |

EQUATIONS

$$-\mathbf{n}\cdot\left(\mathbf{J}_i+\mathbf{u}c_i\right)=0$$

#### Convection

SETTINGS

| Description | Value |
|---|---|
| Include | On |

### Initial Values 1

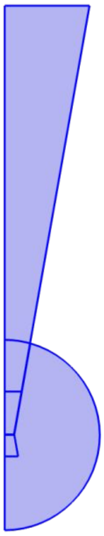

*Initial Values 1*

SELECTION

| | |
|---|---|
| Geometric entity level | Domain |
| Selection | Geometry geom1: Dimension 2: All domains |

### Initial Values

SETTINGS

| **Description** | **Value** |
|---|---|
| Concentration | {cbulk, cbulk} |

## Concentration, bulk solution

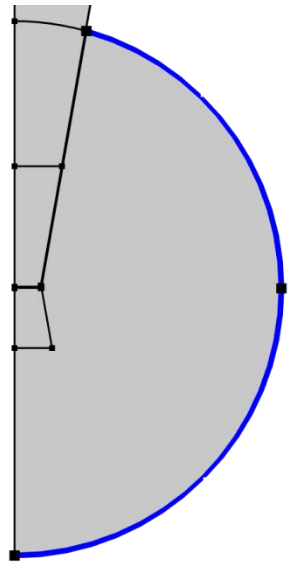

*Concentration, bulk solution*

SELECTION

| | |
|---|---|
| Geometric entity level | Boundary |
| Selection | Geometry geom1: Dimension 1: Boundaries 34, 41–42 |

EQUATIONS

$$c_i = c_{0,j}$$

### Concentration

SETTINGS

| **Description** | **Value** |
|---|---|
| Species cK | On |
| Species cCl | On |
| Concentration | {cbulk, cbulk} |

### Constraint Settings

SETTINGS

| **Description** | **Value** |
|---|---|
| Apply reaction terms on | All physics (symmetric) |
| Use weak constraints | On |

### Concentration, pipette top

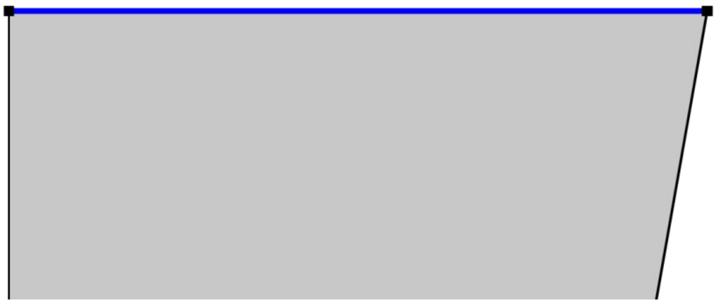

*Concentration, pipette top*

SELECTION

| | |
|---|---|
| Geometric entity level | Boundary |
| Selection | Geometry geom1: Dimension 1: Boundaries 12, 33 |

EQUATIONS

$$c_i = c_{0,j}$$

### Concentration

SETTINGS

| Description | Value |
|---|---|
| Species cK | On |
| Species cCl | On |
| Concentration | {cbulk, cbulk} |

### Constraint Settings

SETTINGS

| Description | Value |
|---|---|
| Apply reaction terms on | All physics (symmetric) |
| Use weak constraints | On |

## CREEPING FLOW 2

USED PRODUCTS

| |
|---|
| Electrochemistry Module |
| COMSOL Multiphysics |

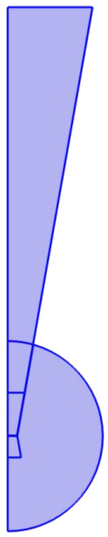

*Creeping Flow 2*

SELECTION

| | |
|---|---|
| Geometric entity level | Domain |
| Selection | Geometry geom1: Dimension 2: All domains |

EQUATIONS

$$0 = \nabla \cdot [-p2\mathbf{I} + \mathbf{K}] + \mathbf{F}$$

$$\rho \nabla \cdot \mathbf{u2} = 0$$

# Interface Settings

## Discretization

SETTINGS

| Description | Value |
|---|---|
| Discretization of fluids | P2 + P1 |

SETTINGS

| Description | Value |
|---|---|
| Equation form | Study controlled |

## Physical Model

SETTINGS

| Description | Value |
|---|---|
| Neglect inertial term (Stokes flow) | On |
| Compressibility | Incompressible flow |
| Enable porous media domains | Off |
| Include gravity | Off |
| Reference temperature | User defined |
| Reference temperature | 293.15[K] |
| Reference pressure level | 1[atm] |

### Turbulence

SETTINGS

| Description | Value |
|---|---|
| Turbulence model type | None |

## Fluid Properties 1

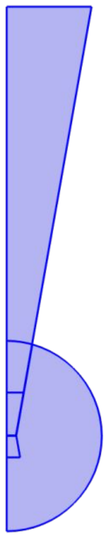

*Fluid Properties 1*

SELECTION

| | |
|---|---|
| Geometric entity level | Domain |
| Selection | Geometry geom1: Dimension 2: All domains |

EQUATIONS

$$0 = \nabla \cdot [-p2\mathbf{I} + \mathbf{K}] + \mathbf{F}$$

$$\rho\nabla \cdot \mathbf{u2} = 0$$

$$\mathbf{K} = \mu\left(\nabla\mathbf{u2} + (\nabla\mathbf{u2})^T\right)$$

### Fluid Properties

SETTINGS

| Description | Value |
|---|---|
| Density | From material |
| | Newtonian |
| Dynamic viscosity | From material |

### Model Input

SETTINGS

| Description | Value |
|---|---|
| Temperature | Common model input |

PROPERTIES FROM MATERIAL

| Property | Material | Property group |
|---|---|---|
| Density | Water, liquid | Basic |
| Dynamic viscosity | Water, liquid | Basic |

## Initial Values 1

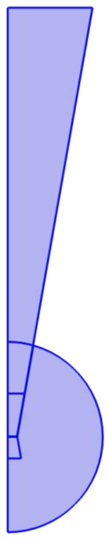

*Initial Values 1*

SELECTION

| | |
|---|---|
| Geometric entity level | Domain |
| Selection | Geometry geom1: Dimension 2: All domains |

### Initial Values

SETTINGS

| Description | Value |
|---|---|
| Velocity field, r component | 0 |
| Velocity field, phi component | 0 |
| Velocity field, z component | 0 |
| Pressure | 0 |

### Coordinate System Selection

SETTINGS

| Description | Value |
|---|---|
| Coordinate system | Global coordinate system |

## Axial Symmetry 1

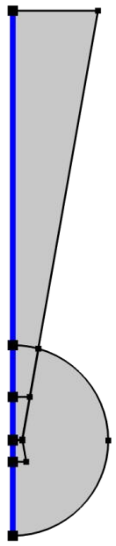

*Axial Symmetry 1*

SELECTION

| | |
|---|---|
| Geometric entity level | Boundary |
| Selection | Geometry geom1: Dimension 1: All boundaries |

## Pipette Wall

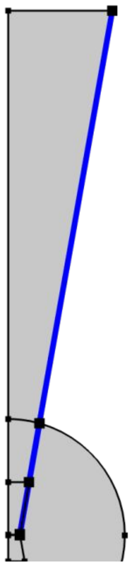

*Pipette Wall*

SELECTION

| | |
|---|---|
| Geometric entity level | Boundary |
| Selection | Geometry geom1: Dimension 1: All boundaries |

EQUATIONS

$$\mathbf{u2} = \mathbf{0}$$

### Boundary Condition

SETTINGS

| Description | Value |
|---|---|
| Wall condition | No slip |

### Wall Movement

SETTINGS

| Description | Value |
|---|---|
| Translational velocity | Automatic from frame |
| Sliding wall | Off |

## Bulk Solution Inlet

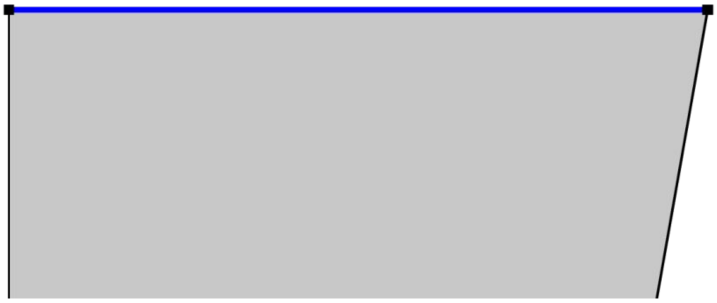

*Bulk Solution Inlet*

SELECTION

| | |
|---|---|
| Geometric entity level | Boundary |
| Selection | Geometry geom1: Dimension 1: Boundaries 12, 33 |

EQUATIONS

$$\mathbf{n}^T[-p2\mathbf{I}+\mathbf{K}]\mathbf{n}=-p_0$$

$$\mathbf{u2}\cdot\mathbf{t}=0$$

### Boundary Condition

SETTINGS

| Description | Value |
|---|---|
| Boundary condition | Pressure |

### Pressure Conditions

SETTINGS

| Description | Value |
|---|---|
| Pressure | Static |
| Pressure | 0 |
| Suppress backflow | Off |
| Flow direction | Normal flow |

## Pipette Top Outlet

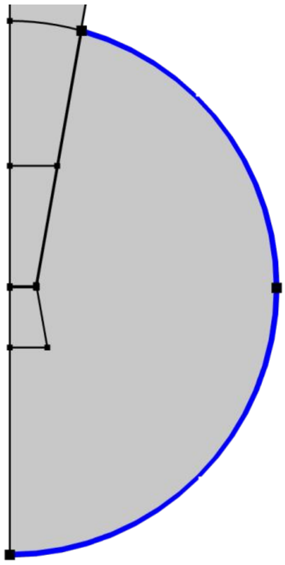

*Pipette Top Outlet*

### SELECTION

| | |
|---|---|
| Geometric entity level | Boundary |
| Selection | Geometry geom1: Dimension 1: Boundaries 34, 41–42 |

### EQUATIONS

$$\mathbf{n}^T[-p2\mathbf{I}+\mathbf{K}]\mathbf{n} = -\hat{p}_0$$

$$\hat{p}_0 \leq p_0, \ \mathbf{u2}\cdot\mathbf{t} = 0$$

## Boundary Condition

### SETTINGS

| Description | Value |
|---|---|
| Boundary condition | Pressure |

## Pressure Conditions

### SETTINGS

| Description | Value |
|---|---|
| Pressure | Static |
| Pressure | 0 |
| Normal flow | On |
| Suppress backflow | On |

## EOF

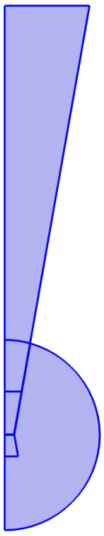

*EOF*

### SELECTION

| | |
|---|---|
| Geometric entity level | Domain |
| Selection | Geometry geom1: Dimension 2: All domains |

### EQUATIONS

$$0 = \nabla \cdot [-p2\mathbf{I} + \mathbf{K}] + \mathbf{F}$$

**Variables**

| Name | Expression | Unit | Description | Selection | Details |
|---|---|---|---|---|---|
| spf2.Fr | es.Er*es.rhoq | $N/m^3$ | Volume force, r component | Domains 1–13 | + operation |
| spf2.Fphi | 0 | $N/m^3$ | Volume force, phi component | Domains 1–13 | + operation |
| spf2.Fz | es.Ez*es.rhoq | $N/m^3$ | Volume force, z component | Domains 1–13 | + operation |

# MULTIPHYSICS

## Potential Coupling 1

### USED PRODUCTS

| |
|---|
| COMSOL Multiphysics |

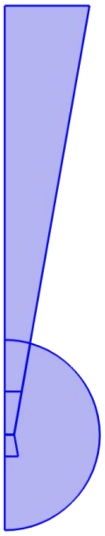

*Potential Coupling 1*

SELECTION

| | |
|---|---|
| Geometric entity level | Domain |
| Selection | Geometry geom1: Dimension 2: All domains |

**Coupled Interfaces**

SETTINGS

| Description | Value |
|---|---|
| Source | Electrostatics (es) |
| Destination | Transport of Diluted Species (tds) |

**Variables**

| Name | Expression | Unit | Description | Selection |
|---|---|---|---|---|
| pc1.V | V | V | Electric potential | Domains 1–13 |

## Space Charge Density Coupling 1

USED PRODUCTS

| |
|---|
| COMSOL Multiphysics |

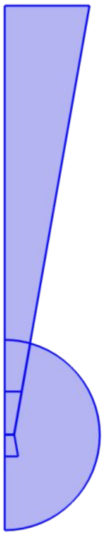

*Space Charge Density Coupling 1*

SELECTION

| | |
|---|---|
| Geometric entity level | Domain |
| Selection | Geometry geom1: Dimension 2: All domains |

EQUATIONS

$$\nabla \cdot \mathbf{D} = \rho_v, \quad \rho_v = F\sum_i z_i c_i$$

**Coupled Interfaces**

SETTINGS

| Description | Value |
|---|---|
| Source | Transport of Diluted Species (tds) |
| Destination | Electrostatics (es) |

## Reacting Flow, Diluted Species 1

USED PRODUCTS

| |
|---|
| COMSOL Multiphysics |

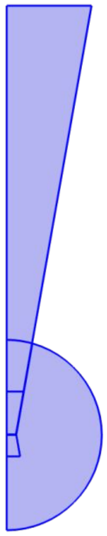

*Reacting Flow, Diluted Species 1*

### SELECTION

| | |
|---|---|
| Geometric entity level | Domain |
| Selection | Geometry geom1: Dimension 2: All domains |

### Coupled Interfaces

#### SETTINGS

| Description | Value |
|---|---|
| Fluid flow | Creeping Flow 2 (spf2) |
| Species transport | Transport of Diluted Species (tds) |

## MESH 1

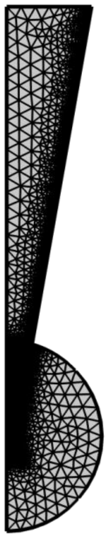

*Mesh 1*

### Size (size)

#### SETTINGS

| Description | Value |
| --- | --- |
| Calibrate for | Fluid dynamics |
| Maximum element size | 1.69E-7 |
| Minimum element size | 7.57E-10 |
| Curvature factor | 0.3 |
| Maximum element growth rate | 1.3 |
| Predefined size | Extra fine |
| Custom element size | Custom |

### Pore mouth vicinity (ftri1)

#### SELECTION

| | |
| --- | --- |
| Geometric entity level | Domain |
| Selection | Geometry geom1: Dimension 2: Domains 2–4, 7–10, 12–13 |

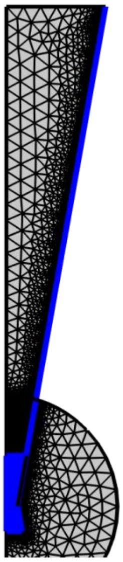

*Pore mouth vicinity*

#### SETTINGS

| Description | Value |
| --- | --- |
| Number of iterations | 4 |
| Maximum element depth to process | 4 |
| Last build time | 2 |
| Built with | COMSOL 6.0.0.312 (win64)\|2022 - 02 - 01T10:40:26.516535200 |

### Cylinder (size1)

#### SELECTION

| | |
| --- | --- |
| Geometric entity level | Domain |
| Selection | Geometry geom1: Dimension 2: Domains 3, 7 |

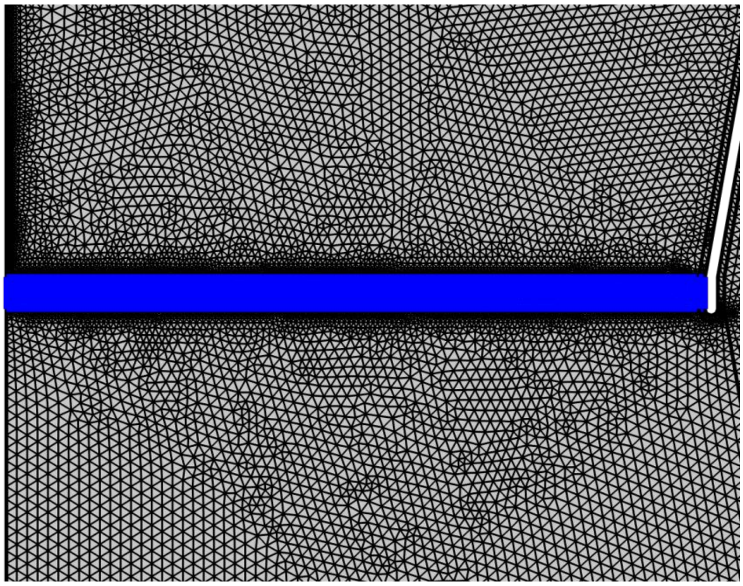

*Cylinder*

SETTINGS

| Description | Value |
|---|---|
| Maximum element size | 0.5e-9 |
| Minimum element size | 1.81E-9 |
| Minimum element size | Off |
| Curvature factor | 0.3 |
| Curvature factor | Off |
| Resolution of narrow regions | Off |
| Maximum element growth rate | 1.3 |
| Maximum element growth rate | Off |
| Custom element size | Custom |

### Size 2 (size2)

SELECTION

| | |
|---|---|
| Geometric entity level | Domain |
| Selection | Geometry geom1: Dimension 2: Domains 2, 4, 8–10 |

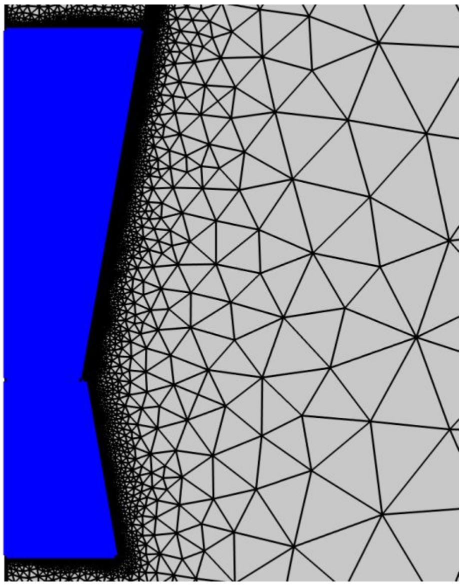

*Size 2*

### SETTINGS

| Description | Value |
|---|---|
| Maximum element size | 2e-9 |
| Minimum element size | 1.81E-9 |
| Minimum element size | Off |
| Curvature factor | 0.3 |
| Curvature factor | Off |
| Resolution of narrow regions | Off |
| Maximum element growth rate | 1.3 |
| Maximum element growth rate | Off |
| Custom element size | Custom |

## Extra nodes for more continous curve extraction (dis1)

### SELECTION

| | |
|---|---|
| Geometric entity level | Boundary |
| Selection | Geometry geom1: Dimension 1: Boundary 7 |

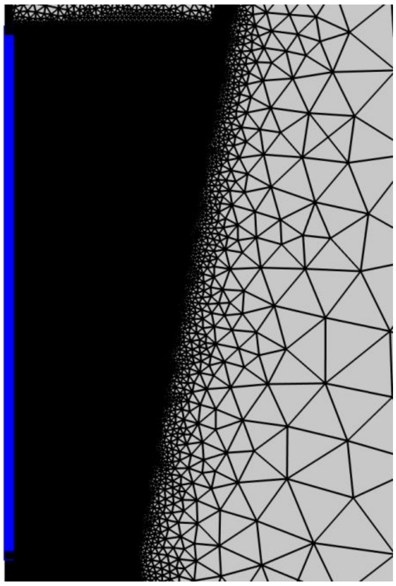

*Extra nodes for more continous curve extraction*

SETTINGS

| Description | Value |
|---|---|
| Number of elements | 1000 |

## Further inside the pipette (ftri2)

SELECTION

| | |
|---|---|
| Geometric entity level | Domain |
| Selection | Geometry geom1: Dimension 2: Domain 5 |

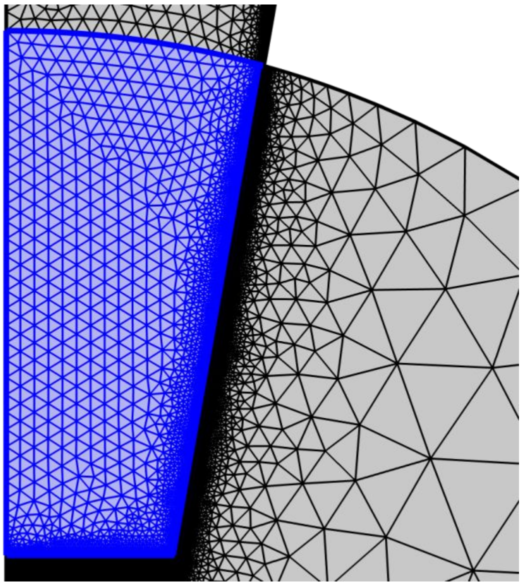

*Further inside the pipette*

SETTINGS

| Description | Value |
|---|---|
| Number of iterations | 4 |
| Maximum element depth to process | 4 |

| Description | Value |
|---|---|
| Last build time | 0 |
| Built with | COMSOL 6.0.0.312 (win64)\|2022 - 02 - 01T10:40:26.619044700 |

### Size 1 (size1)

SELECTION

| | |
|---|---|
| Geometric entity level | Domain |
| Selection | Geometry geom1: Dimension 2: Domain 5 |

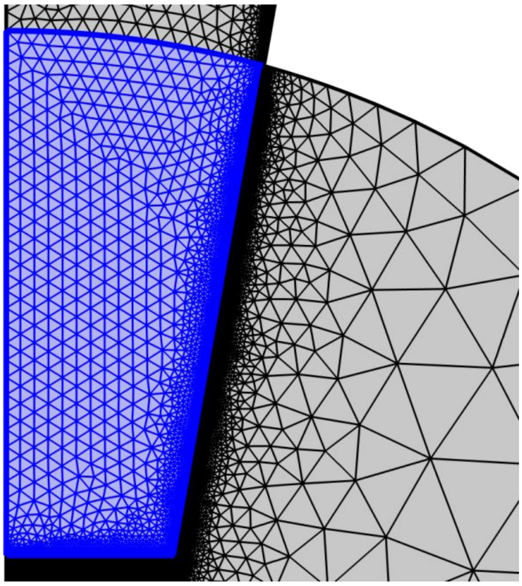

*Size 1*

SETTINGS

| Description | Value |
|---|---|
| Maximum element size | 20e-9 |
| Minimum element size | 1.81E-9 |
| Minimum element size | Off |
| Curvature factor | 0.3 |
| Curvature factor | Off |
| Resolution of narrow regions | Off |
| Maximum element growth rate | 1.3 |
| Maximum element growth rate | Off |
| Custom element size | Custom |

## Interior and exterior bulk (ftri3)

SELECTION

| | |
|---|---|
| Geometric entity level | Domain |
| Selection | Remaining |

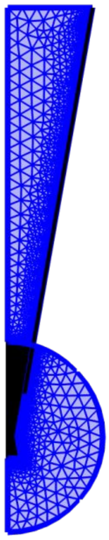

*Interior and exterior bulk*

### SETTINGS

| Description | Value |
|---|---|
| Number of iterations | 4 |
| Maximum element depth to process | 4 |
| Last build time | 2 |
| Built with | COMSOL 6.0.0.312 (win64)\|2022 - 02 - 01T10:40:29.565042800 |
